# The Bad and the Good—Microorganisms in Cultural Heritage Environments—An Update on Biodeterioration and Biotreatment Approaches

**DOI:** 10.3390/ma14010177

**Published:** 2021-01-01

**Authors:** Adam Pyzik, Karol Ciuchcinski, Mikolaj Dziurzynski, Lukasz Dziewit

**Affiliations:** Department of Environmental Microbiology and Biotechnology, Institute of Microbiology, Faculty of Biology, University of Warsaw, Miecznikowa 1, 02-096 Warsaw, Poland; k.ciuchcinski@student.uw.edu.pl (K.C.); mikolaj.dziurzynski@biol.uw.edu.pl (M.D.); ldziewit@biol.uw.edu.pl (L.D.)

**Keywords:** bacteria, fungi, biodeterioration, biotreatment, meta-omics, cultural heritage

## Abstract

Cultural heritage objects constitute a very diverse environment, inhabited by various bacteria and fungi. The impact of these microorganisms on the degradation of artworks is undeniable, but at the same time, some of them may be applied for the efficient biotreatment of cultural heritage assets. Interventions with microorganisms have been proven to be useful in restoration of artworks, when classical chemical and mechanical methods fail or produce poor or short-term effects. The path to understanding the impact of microbes on historical objects relies mostly on multidisciplinary approaches, combining novel meta-omic technologies with classical cultivation experiments, and physico-chemical characterization of artworks. In particular, the development of metabolomic- and metatranscriptomic-based analyses associated with metagenomic studies may significantly increase our understanding of the microbial processes occurring on different materials and under various environmental conditions. Moreover, the progress in environmental microbiology and biotechnology may enable more effective application of microorganisms in the biotreatment of historical objects, creating an alternative to highly invasive chemical and mechanical methods.

## 1. Introduction

Cultural heritage objects represent highly heterologous habitats, both in terms of the microbiome’s structure and composition. Even though they are mostly considered as oligotrophic, these habitats can be colonized by various groups of microorganisms. Artworks made from textiles, paper, wood and even stones provide substrates for microbial growth, which often go unnoticed unless biofilm overgrowth and discoloration or weakening of the physical integrity of the material occurs. In consequence, many objects may lose some of their cultural and monetary value.

Much has been done to study the diversity of microorganisms responsible for biodeterioration, but there are still many unanswered questions. This lack of knowledge inhibits the development of effective and non-destructive conservation strategies. Since conventional physico-chemical methods are often ineffective and dangerous for treated artworks (as well as people involved in the conservation process), new strategies are constantly being developed to mitigate the negative impact of microorganisms. These include the use of gamma irradiation [1], vaporized hydrogen peroxide [2], low- temperature helium-generated plasm [3], volatile compounds and essential oils [4] as well as various natural biocides obtained from microorganisms, marine organisms or plants [5]. Many of them still need to be further verified, testing their usability in different study-case scenarios and with various materials. Nowadays, biocides are commonly used to limit biodeterioration of various objects. Amongst them, the application of nanoparticles is gaining increasing attention [5,6]. However, due to the highly penetrative nature of nanoparticles and their long-lasting effect on living organisms as well as their unknown interference with the treated material, their usage must be carefully monitored [7].

When historical artworks become damaged, intervention and conservation techniques need to be applied. A promising alternative for traditional strategies is the use of natural processes performed by microorganisms. Compounds of biological origin, such as calcium carbonate, can be used for biotreatment of damaged stones [8], while calcium oxalate films may act as an additional protective layer [9]. Biological treatment is characterized by low cost and low invasiveness, as well as high specificity and easy control when compared to traditional physico-chemical techniques [10]. Due to the multi- component nature of the decay, a combination of biological treatment and various other methods was also tested, with initial gentle treatment by mechanical, chemical or laser-based methods followed by the main cleaning process using microorganisms [11,12,13].

An important issue in cultural heritage microbiology is also public health concern. International human traffic within museums makes cultural heritage estates similar to some of the most crowded airports. In 2019, almost the same number of visitors (approximately 28 million) was recorded for the top five London museums combined (British Museum, Tate Modern, National Gallery, Natural History Museum, Victoria & Albert Museum) as for London Stansted Airport [14]. These make cultural heritage objects important bridge-nodes in the global network of pathogen spread. Therefore, the significance of such objects in pathogen transmission must not be underestimated and has to be further evaluated [15,16,17].

## 2. Inspection of Methodologies Applied for Identification and Characterization of Microorganisms in Cultural Heritage Objects

**Figure 1:**
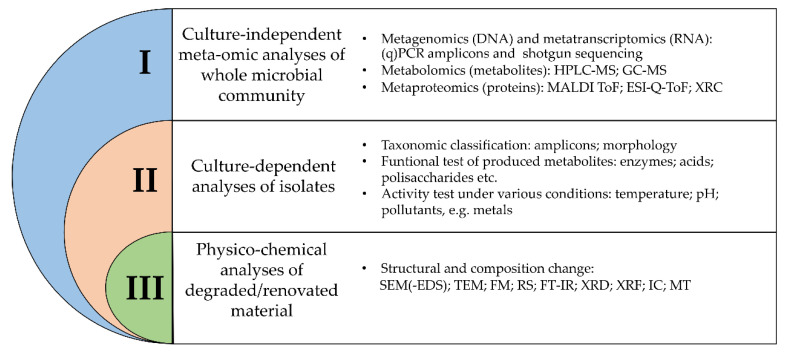
Overview of different methodologies for the study of microbial communities in relation to their impact on organic and inorganic materials. (q)PCR—(quantitative) polymerase chain reaction; HPLC-MS—liquid chromatography-mass spectrometry; GC-MS—gas chromatography-mass spectrometry; MALDI ToF—matrix-assisted laser desorption/ionization time-of-flight; ESI-Q-ToF—electrospray-ionization quadrupole time-of-flight mass spectrometry; XRC—X-ray crystallography; SEM(-EDS)—scanning electron microscopy (coupled with energy-dispersive X-ray spectroscopy); TEM—transmission electron microscopy; FM –fluorescence microscopy; RS—Raman spectroscopy; FT-IR—Fourier transform coupled infrared spectroscopy; XRD—X-ray diffraction; XRF—X-ray fluorescence; IC—ion chromatography; MT—microtomography.

When studying objects of cultural heritage, three levels of complex microbiological research can be distinguished (Figure 1). Integration of all three approaches may provide a better understanding of microorganisms (including biodetriogens) inhabiting and influencing historical and cultural objects [18].

Selection of an appropriate methodology for microbiological research is usually based on conservation restrictions and study purpose, such as general contamination (biodeterioration and human health risk assessment) or targeted research on a given artwork/material [19,20,21,22,23]. For general contamination assessment, the air is the most popular study subject, therefore numerous sampling techniques have been developed with (i) impaction, (ii) filtration, and (iii) impingement as major methods [24]. Microorganisms are collected onto an agar medium, filter and liquid medium, respectively. While impaction is a strictly culture-based approach, the other two may be culture-independent.

Although each method has its benefits, they also have their drawbacks. For example, the use of the culture-based approach, coupled with impaction, introduces a serious bias in the general biodiversity recovery rate. This is due to two main factors, i.e., the cultivation-based approach bias and the fact that if the agar medium is too solid, the cells can simply bounce back. The main drawback of the filtration method is low recovery efficiency and filter overloading [25]. The main disadvantage of impingementation is related to the evaporation of liquid. However, this may sometimes benefit the experiment, as it increases the concentration of the sample before the subsequent analyses [24,26].

When research on historic surfaces is conducted, only non-invasive or micro-invasive treatment methods can be applied using sterile scalpels, swabs, membrane filters or adhesive tapes [22,23,27]. However, it should be noted that the use of various sampling methods can provide different results. For example, nylon membranes are more reliable than cotton swabs, as the latter tend to overestimate the surface microbiota, which may not be involved in the biodeterioration process [27]. This stems from the fact that microbes tend to become endolytic in the search of nutrients but also need to protect themselves from UV radiation, temperature fluctuations and desiccation [28,29,30,31].

### 2.1. Culture-Independent Meta-Omic Analyses

Nowadays, metagenomic studies are often applied for the estimation of microbial contamination of objects of cultural heritage. Usually, the diversity of bacteria and fungi is analyzed by amplicon sequencing (metabarcoding), as this method requires a smaller amount of DNA and is cheaper than the whole metagenome sequencing.

The most common taxonomic markers are fragments of genes encoding ribosomal DNA (rDNA). In the case of bacteria, in particular, V3 and V4 hypervariable regions of the 16S rDNA gene are commonly used, as they provide the most reliable results [32,33,34]. In comparison, fungal diversity is usually studied using internal transcribed spacers (ITS), either ITS1 or ITS2. In general, the use of the ITS2 region is preferred as it was shown that the use of ITS1 can lead to overestimation of diversity and richness when compared to the ITS2 region [35]. However, a recent study showed that in the case of bio-aerosols the ITS1 region might be more suitable [36]. To avoid additional bias, such as chimeric and overrepresented sequences, the number of PCR cycles should be minimized and the use of proofreading DNA polymerase is highly recommended [37]. A maximum of thirty cycles of PCR should be sufficient for culture-dependent analyses, as well as culture- independent analyses of air and dust microbiomes.

There are many tools for the assessment of biodiversity based on amplicons. For example, the QIIME2 platform is extensively used for most bioinformatic analyses involved in the process of biodiversity research [38]. Various amplicon analysis tools have been already compared and reviewed elsewhere [39,40], but it is important to note that workflows based on Amplicon Sequence Variant (ASV), employed by DADA2, USEARCH-UNOISE3, and QIIME2, seem to be superior to the ones based on Operational Taxonomic Units.

Relatively easy, fast, reliable, and cost-effective quantitative assessment of microbial contamination could also be done by qPCR, targeting 16S rDNA gene, ITS regions or *rbcl*, *tufA* and *actB* genes [41,42,43]. Amplification primers, used both in PCR and qPCR reactions, usually cover specific groups of microorganisms, but degenerate primers, applicable to a broad range of taxa are used as well [42]. As mentioned earlier, in the case of bio-aerosols, a loss of material often occurs during sample processing, thus spiking and normalization of measurements needs to be done, allowing good correlations between qPCR and high- throughput sequencing [42].

A significant development in metagenomic studies came with the introduction of third-generation sequencing technologies. Most commonly used short-read based technologies (<300 bp) enable reliable biodiversity investigation on the genus level at the maximum, while third-generation sequencing technologies make it possible to analyze the data on species or even the strain level due to longer sequencing reads [44]. Recent researches show that many species-level assignments could be done only with long-read sequences, thus indicating the importance of improved resolution for accurate assessment of degradation/pathogenicity potential of environmental samples, usually observed for certain species or strains, but not always on the genera level [23,45].

Detailed characterization of microbial communities thriving on historical assets (in terms of both diversity and metabolic functions) can be done by whole metagenome sequencing [46,47]. However, this approach is rarely used in cultural heritage due to strict requirements of non-invasive or minimal-invasive sampling, which often prevents the required amount of DNA from being collected. Among available technologies, MinION devices (Oxford Nanopore system) exhibit high potential for application in the cultural heritage area as they require only a small amount of DNA. The small size of the device makes it suitable for on-site analyses. Moreover, longer reads gave a better insight into the relative proportions and metabolic potential of studied microbiomes [46]. The combination of various sequencing methods suitable for whole metagenome sequencing, such as the Oxford Nanopore, providing long reads but with lower accuracy when compared to the short but highly accurate reads provided by the Illumina MiSeq system, can significantly increase genome coverage (~200×) and therefore the quality of metagenome-derived genomes [48].

Although the majority of research related to cultural heritage microbiology is based on DNA sequencing, recent reports indicate that diversity analyses based on RNA molecules may provide better results as they focus on the identification of active microbes, rather than the overall microbial community [49]. Nevertheless, it should be noted that microorganisms often shift between the dormant and active state, depending on the environmental conditions, thus, when carrying out RNA-based analyses, sampling should be conducted multiple times, to prevent time-point bias of complex microbial communities [50,51]. RNA-based research is still rarely performed in cultural heritage microbiology, but there are already some interesting examples of total bacterial transcriptome analyses associated with historic artworks [52]. It is important to mention that one of the limitations of metatranscriptomic analyses is the lack of well-developed databases, but this may be overcome if metagenome-guided research is performed in parallel. In such a situation, metatranscriptomics can significantly contribute to the understanding of microbial processes occurring in the studied environment [53].

Among meta-omic approaches, other techniques have been recently introduced into the cultural heritage field. These novel methods are still rarely used and include metabolomic studies of wood and brick [54], wax seals [55], photographs [56], as well as metaproteomic studies of books [57], historical wood, frescoes and canvases [58,59], mummies and documents [60]. The metabolomic approach allows the investigation of many different compounds as it can detect and analyze proteins, lipids, and carbohydrates. Metabolomic studies may be either targeted, allowing for the analysis of a set of individual metabolites such as pigments, or untargeted (global), providing an insight into thousands of metabolites, which can be useful for screening for biomarkers of biological processes, such as biodeterioration.

### 2.2. Culture-Dependent Analyses

In cultural heritage assets, the most commonly used approach for monitoring of microorganisms is based on culture-dependent methods. Classical cultivation on agar media is simple, cheap, and can be easily applied by the basic-trained personnel belonging to museums. There are many different types of media with some specificity towards different taxonomic groups [61,62,63]. The main disadvantage of this approach is that the majority of microorganisms are not cultivable on classical media, which neglects the importance of viable but not culturable microorganisms [64]. Furthermore, while rich media are commonly used, they are not suitable for the overall biodiversity analyses [65]. Nevertheless, a cultivation approach can provide important data concerning microbial phenotypes, especially when prolonged incubation periods and low-substrate media are used for the increased recovery of environmental isolates [66,67]. Additionally, meta- omics aided cultivation strategies are promising for the isolation of yet uncultured microorganisms [68].

An undoubtful advantage of cultivable techniques is the enrichment of several microbial strains obtained in high cell density, which is crucial to perform laboratory assays (simulation assays or mitigation assays. Physiological analyses of single isolates may provide insight into the metabolic potential of a given microorganism (biodeteriogen), including its ability for pigment production, acid/alkaline production, proteolytic/cellulolytic activity or carbonate dissolution [69]. For example, in the work of Pavić and colleagues, it was shown that cultivable biodeteriogens were able to produce proteases, esterases, and lipases and grew on dyes as a sole source of phosphate and iron [70].

### 2.3. Physico-Chemical Analyses

Analyses of microorganisms associated with biodeterioration or biotreatment processes are more meaningful if auxiliary techniques, which visualize structural modifications or chemical changes, are applied. It has already been shown, that for complex multi-component analyses several commonly acknowledged techniques may be combined, e.g.,: (i) XRF, XRD, SEM, FM, isotope analyses, coupled with cultivation experiments on Reasoner’s 2A agar and 16S rDNA analyses (Applied Biosystems platform) [71]; (ii) SEM, RS, FTIR, together with cultivation experiments on Mueller–Hinton medium and 16S rDNA analyses (Applied Biosystems platform) [72]; (iii) XRD, RS, FTIR, SEM-EDS, confocal microscopy, accompanied with transcriptomic sequencing (Illumina) and shotgun sequencing (PacBio) [52].

Nowadays, some novel, non-invasive, analytical chemistry techniques are being applied in cultural heritage analyses. FTIR microspectroscopy and Raman spectroscopy are examples of non-destructive methods gaining increasing attention. These methods enable the monitoring of microbial contamination and the state of artwork by spectra identification. They have already been employed for the analyses of pigment [72], textile fibers [73,74], mineral formation [52], and other natural organic substances such as oils, gums or glues [75]. One of the newest methodologies, allowing for early detection of microorganism activity is a technique using RGB-ITR laser scanner (Red Green Blue Imaging Topological Radar) [76]. This approach is based on laser-induced fluorescence and 3D digitalization of the site and it can detect changes even in areas where biodeterioration is not yet evident.

The activity of microbes on artworks may be also assessed by ATP and FDA assays [77,78,79]. However, these activity assays should be applied only for rapid and simple testing as a preliminary evaluation before further analyses are performed [78].

## 3. Microorganism-Driven Deterioration of Historical Artefacts

Bacteria and fungi have significant potential to negatively affect historic artworks (Table 1) [31,80,81,82,83]. There are guidelines to preserve indoor cultural heritage artworks [84] with humidity control as one of the major factors affecting the viability of microorganisms, especially fungi [85]. Furthermore, indoor environment microbiome can be altered by architectural design, ventilation strategies, and occupancy patterns [86] and have been proven to be highly affected by human occupancy [87]. In contrast, outdoor artworks (mainly stones) are more exposed to deterioration processes due to changing environmental conditions and the lack of effective prevention strategies.

Outdoor stone artworks are highly specific for biodeterioration investigations, as they usually harbour so-called subaerial biofilms-mixed, phototroph-heterotroph based communities that are highly dependent on surface/air interface conditions [88]. Although this relation has been modelled in the past, the dependence of microbial communities on environmental conditions has not been fully elucidated, especially when bacterial and fungal communities interact and form complex biofilms which could be considered as one meta-organism [89]. Development of biofilms, built from extracellular polymeric substances (EPS; such as polysaccharides, lipids, proteins, nucleic acids, pigments, and enzymes), significantly amplify the biodeterioration processes of various materials, while conferring increased resistance to biocidal compounds. Furthermore, biofilms also mediate increased recovery of nutrients due to interspecies interactions, as well as by entrapment of airborne particles of organic and inorganic compounds.

Understanding biodeterioration processes of cultural heritage artworks requires repeated, long term monitoring to avoid misleading conclusions. For example, Leplat and colleagues confirmed a rosy discoloration of stones of bacterial origin, but within the three years of research, they highlighted that pigmentation was already finished when the study was started [90]. It is also important to remember that biodeterioration may happen due to the indirect action of compounds originating from previous activity of the microorganisms [27].

Among microorganisms named as biodeteriogens of cultural heritage, many bacteria and fungi have been recognized as key aggressors (Table 1). Often the same species is involved in the degradation of different materials, but deterioration is usually caused by the action of multiple types of microorganisms that form biofilms. For example, *Streptomyces* spp. are involved in the deterioration of cultural heritage materials through several pathways, with pigment production as one of the most important [100]. Similarly, *Aspergillus* sp. and *Penicillium* sp. are effective producers of pigments, but also different extracellular enzymes and acids, as well as physical damage contribute significantly to structural alterations of cultural heritage materials [69,101]. Taking into account the type of material, the different microbiological requirements can be distinguished. Stone and metal materials are first colonized by autotrophic (e.g., *Eucapsis* sp.) and lithotrophic (e.g., *Desulfovibrio* sp.) organisms and then by heterotrophic microbes such as *Sphingomonas* sp. or *Penicillium* sp. In contrast, other materials are mainly colonized by heterotrophs that exhibit different enzymatic activities such as lignocellulolytic activity in the degradation of wood and paper (e.g., *Clostridium* sp., *Aspergillus* sp.); cellulolytic, keratinolytic, and esterase-producing activity in the degradation of textiles (e.g., *Bacillus* sp., *Trichoderma* sp.); lipolytic, amylolytic, proteolytic and solventogenic activity in the degradation of paintings and photographs (e.g., *Pseudomonas* sp., *Cladosporium* sp.) [81].

Application of high-throughput sequencing has shown that many artworks are inhabited by numerous unclassified bacteria and fungi, suggesting that there are various unknown microorganisms, yet to be included on the bioderiogens’ list [94]. New microorganisms are constantly being recognized as important players in cultural heritage deterioration, such as *Stenotrophomonas tumulicola* [102], *Myxacorys almedinensis* [103], *Lecanicillium gracile* [104], *Periconia epilithographicola* [105], *Coniochaeta cipronana* [105]; *Aeminium* spp. *and Aeminium ludgeri* spp. [106]. Additionally, the role of already known microbes in biodeterioration processes is also being constantly revised, e.g., *Parengyodontium album* [107]. The abovementioned microbes exhibit xerophilic or halophilic, or cellulolytic activity adaptations, forming biofilms or producing pigments. These features allow them to grow and preserve activity on historical artworks, leading to structural alterations.

Among various materials, cellulose-made cultural heritage objects (mainly wood and paper) are the most accessible nutrient source for bacteria and fungi. Degradation of waterlogged and buried wood is much slower than for that found on land, but once excavated, decay can occur very rapidly [108]. Among known microorganisms contributing to the degradation of historical objects, cellulolytic bacteria such as *Cellulomonas*, *Cellvibrio*, *Clostridium,* and fungi *Chateomium* and *Fusarium* are often mentioned (Table 1).

Canvas paintings are the most nutrient-rich artworks, as in addition to wood and fabrics they also contain organic pigments, resins, and solvents, which can be utilized by various lipolytic, amylolytic, and proteolytic microorganisms [81]. Fungi belonging to the genus *Aspergillus* and bacteria of the order Burkholderiales were indicated to be major biodeteriogens of damaged paintings [46].

Mural paintings are habitats with limited nutrient content compared to canvas paintings, however they are still colonized by microorganisms such as *Gloeocapsa* sp., *Rubrobacter* sp., *Aspergillus* sp., and *Penicillium* sp. [72,95,96,97,109]. These microorganisms are among many others which contribute to the biodeterioration processes, manifested as discoloration, bulging plaster, or peeling pictorial layer.

In comparison to paintings, stone objects seem to have few nutrients, but many microorganisms have still adapted to utilize minerals found on these surfaces, compounds deposited from the air, as well as NO_2_ and SO_2_, which provide nutrition for nitrifying bacteria (e.g., *Nitrosomonas* and *Nitrobacter*) and sulfur-oxidating bacteria (e.g., *Thiobacillus*) [80]. The presence of microorganisms such as *Rubrobacter*, *Arthrobacter*, *Roseomonas*, and *Marinobacter* seems to be responsible for colored biofilm formation while *Ulocladium*, *Cladosporium*, and *Dirina* may be related to structural damage [110]. Li and colleagues analyzed co-occurrence networks allowing the interaction between core bacteria and stone deterioration to be understood. They indicated *Bryobacter*, *Chroococcidiopsis*, *Rubrobacter*, *Blastocatella*, *Sphingomonas,* and *Loriellopsis* as the most important deteriogens [96]. Another study indicated stone-type driven adaptations, where granite-inhabiting bacteria increased the abundance of genes relevant to acid- tolerance and chemotaxis, while limestone-associated bacteria have more photosynthesis- and radiation-resistance-related genes [111].

The role of Fungi and Bacteria domains is well studied in cultural heritage but recently attention has been given also to Archaea as their ammonia-oxidizing representatives were shown to be involved in the biodeterioration of stones [112,113].

## 4. Biotechnological Use of Microorganisms in Cultural Heritage

Microorganisms can be used for the biorestoration of various artworks (Table 2). As of now, living cells are still being applied more often than their metabolites, but the metabolites alone can be also used [114]. Enzymatic treatment in cultural heritage restoration is currently limited because of its poor efficiency and low operational and environmental stability. Interestingly, some modifications, such as enzyme immobilization on gold nanoparticles, have been shown to improve the efficacy of enzymatic treatment by increasing its resistance to environmental conditions up to 5-fold [115]. Biological treatment is constantly being developed as its use may depend on the material processed.

Active biorestoration methods applying microorganisms are commonly being used for the removal of glue from paper and textiles, nitrate or sulfate salt crust removal or bioconsolidation of stones [10,133]. Importantly, bacteria are used more often than fungi in cultural heritage conservation–intervention processes. The most commonly used bacterial strains are: (i) *Desulfovibrio vulgaris*—for the removal of sulfates, *Pseudomonas stutzeri*—for the removal of nitrogen salts and organic matter and *Bacillus spp.*—for bioconsolidation through calcium carbonate production [10].

The most studied, bio-based treatment is bioconsolidation of outdoor stone monuments. The bioconsolidation mechanism is based on calcium carbonate precipitation which may occur through different pathways, i.e., (i) sulfate reduction (*Desulfovibrio* sp.), (ii) ammonification (*Myxoccoccus* sp.), (iii) denitrification (*Pseudomonas* sp.), (iv) conversion of organic acids (*Bacillus* sp.) and (v) most commonly ureolysis (*Sporosarcina* sp., *Bacillus* sp.). Nevertheless, ureolysis seems to be the most efficient strategy thus is the most often applied [134,135]. An important issue in biotreatment is also nitrate or sulfate salt crust removal from mural paintings, as the presence of these salts contributes significantly to physical and aesthetical damage to the artworks [116,118,122]. Biotreatment is often performed using *Pseudomonas* sp. and *Desulfovibrio* sp. [136].

Successful application of bacteria for biotreatment of different materials (Table 2), was shown i.e., for *Thiobacillus denitrificans* for FeS and mineral sulfur transformation for historical wood treatment [131] or *Pseudomonas stutzeri*, *Aerobacter aerogenes*, *Comamonas* sp. for graffiti cleaning [132]. Animal glues were often used in paper manufacturing and reparation of ancient paper artworks, but aged glue can create distortions, tensions, cockling and discoloration of paper materials. Thus, its removal is essential for the preservation of valuable documents. Enzymatic mixtures produced by *Paracoccus* sp., *Bacillus flexus*, *Exiguobacterium undae* [130], as well as the use of *Ochrobactrum* sp. TNS15 [129] gave a very promising result in removing the glue layer without damaging the paper or leaving undesirable residues.

Another example in which biotreatment is used, concerns corroded metal objects. To protect excavated items from corrosion, bacterial strain *Desulfitobacterium hafniense* TCE1 was successfully used to reduce iron, which led to stabilization of archaeological iron by the formation of biogenic vivianite and magnetite on the surface of the artefacts [126]. Corroded metal is one of the few examples for which fungi has been used for biorestoration of historical artworks. Albini and colleagues showed a novel method for inhibition of corrosion of copper surfaces. The method is based on biopassivation resulting from the action of fungus *Beauveria bassiana* (eco-friendly non-toxic). This method proved to be more efficient than the traditional use of benzotriazole, while being safe for the environment and personnel, as opposed to benzotriazole [137]. The second example of fungi being used in the cultural heritage field is for the removal of starch paste adhesives from historical textiles using α-amylase enzyme produced by *Aspergillus oryzae* [138].

It needs to be noted, that fungi usually exhibit a more devastating effect on cultural heritage objects than bacteria. Interestingly, for the protection of cultural heritage assets against fungi, *Bacillus*-based treatments have been proposed [139,140]. This is an example of how “good” microbes can be directly used against biodeteriogens.

## 5. Conclusions

This review summarizes research on biodeterioration and biological treatment, focusing on the latest methodology that enables a better understanding of the influence of microbes on cultural heritage artworks. We emphasized that the multidisciplinary approach allows for formulation of more effective application strategies in cultural heritage environments that favor the protection and longevity of historical artworks. To reduce the bias while obtaining targeted results, the best approach is to combine in tandem a culture-dependent (cultivation) and culture-independent (meta-omics) approach to identify microorganisms and metabolites that are harmful to a given type of artwork, e.g., stone, wood, or metal materials. The best practice for artwork sampling should include the analysis of the epilytic, but even more the endolytic, microorganisms from a representative degraded object. In order to control biodegradation processes, it is necessary to repeatedly take samples and/or formulate a co-occurrence network of significantly interacting microbes. In addition, a correlation between biodiversity (optimally based on the RNA molecule) and the environmental factors and compounds of a given material is desirable. It can be difficult to carry out all analyses in one project, but data on specific aspects of biodegradation or biological treatment may provide a collective understanding of the ongoing processes.

## Figures and Tables

**Table 1:** Microorganisms associated with biodeterioration of various materials.

Material	Bacteria	Fungi	Reference
Textile and canvas paintings	*Achromobacter* sp., *Alcaligenes* sp., *Arthrobacter* sp., *Bacillus* sp., *Brevibacterium* sp., *Cellulomonas* sp., *Cellvibrio* sp., *Cellfalcicula* sp., *Clostridium* sp., *Corynebacterium* sp., *Cytophaga* sp., *Halobacillus* sp., *Kocuria* sp., *Micrococcus* sp., *Microspora* sp., *Microbispora* sp., *Myxococcoides* sp., *Nocardia* sp., *Oceanobacillus* sp., *Paracoccus* sp., *Paenisporosarcina* sp., *Pseudomonas* sp., *Proteus* sp., *Rhodococcus* sp., *Sporocytophaga* sp., *Staphylococcus* sp., *Streptomyces* sp.	*Acremonium* sp.*, Alternaria* sp.*, Aspergillus* sp.*, Aureobasidium* sp.*, Candida* sp.*, Cephalothecium* sp.*, Chaetomium* sp.*, Chrysosporium* sp.*, Cladosporium* sp.*, Epidermophyton* sp.*, Fusarium* sp.*, Microsporum* sp.*, Mucor* sp.*, Myrothecium* sp.*, Neurospora* sp.*, Penicillium* sp.*, Rhizopus* sp.*, Scopulariopsis* sp.*, Stachybotrys* sp.*, Stemphylium* sp.*, Talaromyces* sp., *Tolypocladium* sp.*, Trichoderma* sp.*, Trichophyton* sp.*, Uloclodium* sp.*, Verticillium* sp.	[29,81,82,83,91]
Wood, paper, parchment	*Bacillus* sp.*, Cellulomonas* sp.*, Cellvibrio* sp.*, Cellfacicula* sp., *Clostridium* sp*., Cytophaga* sp.*, Marinobacter* sp.*, Micromonospora* sp.*, Xanthomonas* sp.*, Pseudomonas* sp.*, Sphingomonas* sp.*, Staphylococcus* sp.*, Virgibacillus* sp.	*Acremonium* sp.*, Alternaria* sp.*, Antrodia* sp., *Aspergillus* sp.*, Aureobasidium* sp.*, Chaetomium* sp., *Chrysosporium* sp.*, Cladosporium* sp., *Coniophora* sp.*, Coriolellus* sp.*, Donkioporia* sp.*, Epicoccum* sp., *Fibuloporia* sp.*, Fusarium* sp*., Gloeophyllum* sp.*, Gymnoascus* sp., *Mucor* sp.*, Paecilomyces* sp.*, Penicillium* sp., *Rhizopus* sp.*, Serpula* sp.*, Trichoderma* sp., *Verticillium* sp.	[81,82,92,93]
Stone and wall paintings	*Alcaligenes* sp.*, Arthrobacter* sp.*, Bacillus* sp.*, Blastococcus* sp.*, Blastocatella* sp.*, Bryobacter* sp.*, Chroococcidiopsis* sp.*, Eucapsis* sp.*, Flavobacterium* sp.*, Kocuria* sp.*, Leptolyngbya* sp.*, Micrococcus* sp.*, Modestobacter* sp.*, Mycobacterium* sp.*, Nitrobacter* sp.*, Nocardia* sp.*, Paenibacillus* sp.*, Pseudomonas* sp.*, Pseudonocardia* sp. *Rubrobacter* sp.*, Sarcina* sp.*, Scytonema* sp.*, Sphingomonas* sp.*, Staphylococcus* sp.*, Streptomyces* sp.*, Thiobacillus* sp.	*Alternaria* sp., *Aspergillus* sp.*, Capnobotryella* sp.*, Cladosporium* sp., *Coniosporium* sp.*, Exophiala* sp. *Hortea* sp.*, Knufia* sp.*, Mucor* sp.*, Trimmatostroma* sp., *Sarcinomyces* sp.*, Penicillium* sp., *Rhizopus* sp.*, Serpula* sp.*, Trichoderma* sp.	[80,81,93,94,95,96,97]
Metal	*Acinetobacter* sp.*, Chryseobacterium* sp.*, Desulfomicrobium* sp, *Desulfosarcina* sp., *Desulfovibrio* sp.*, Methanococcus* sp., *Shewanella* sp., *Sphingomonas* sp.*, Stenotrophomonas* sp.*, Thiobacillus* sp*, Vibrio* sp.	*Alternaria* sp., *Antrodia* sp.*, Arthrinium* sp.*, Aspergillus* sp.*, Candida* sp., *Cladosporium* sp.*, Clonostachys* sp.*, Cryptococcus* sp.*, Chrysosporium* sp.*, Debaryomyces* sp.*, Exophiala* sp.*, Fusarium* sp.*, Paecilomyces* sp.*, Penicillium* sp.*, Pichia* sp., *Poria* sp.*, Rhodotorula* sp.	[98,99]
Photographs	*Burkholderia* sp.*, Delftia* sp.*, Enhydrobacter* sp.*, Mesorhizobium* sp., *Neisseria* sp., *Olsenella* sp., *Paenibacillus* sp.*, Pseudomonas* sp., *Saccharopolyspora* sp.	*Alternaria* sp.*, Aspergillus* sp.*, Chaetomium* sp.*, Fusarium* sp.*, Penicillium* sp.*, Talaromyces* sp.*, Trichoderma* sp.	[56,81]

**Table 2:** Examples of bacteria applied in biotreatment of cultural heritage objects.

Material	Bacteria	References
Stone and wall paintings	The indigenous community of carbonatogenic bacteria, *Acinetobacter* sp., *Bacillus* sp., *Brevibacterium* sp., *Cupriavidus metalidurans*, *Cellulosimicrobium cellulans, Desulfovibrio desulfuricans*, *Desulfovibrio vulgaris*, *Halomonas campaniensis*, *Micrococcus* sp., *Myxococcus xanthus*, *Pantonea* sp., *Pseudomonas aeruginosa*, *Pseudomonas chlororaphis*, *Pseudomonas koreensis*, *Pseudomonas stutzeri*, *Sporosarcina pasteurii, Stenotrophomonas maltophilia*	[116,117,118,119,120,121,122,123,124,125]
Metal	*Aeromonas* sp., *Desulfitobacterium hafniense*, *Geobacter sulfurreducens*, *Shewanella loihica*, *Sporosarcina pasteurii*	[52,126,127,128]
Paper/glue	*Bacillus flexus*, *Exiguobacterium undae*, *Ochrobactrum* sp., *Paracoccus* sp.	[129,130]
Textile	*Bacillus* sp.	[115]
Wood	*Thiobacillus denitrificans*	[131]
Graffiti	*Aerobacter aerogenes*, *Comamonas* sp., *Pseudomonas stutzeri*	[132]

## Data Availability

Data sharing not applicable.
